# SPOP the mutation

**DOI:** 10.7554/eLife.11760

**Published:** 2015-10-27

**Authors:** Leah Rider, Scott D Cramer

**Affiliations:** Department of Pharmacology and Molecular Sciences, University of Colorado, Aurora, United States; Department of Pharmacology, University of Colorado, Aurora, United Statesscott.cramer@ucdenver.edu

**Keywords:** prostate cancer, cancer genomics, SPOP, DNA repair, human, mouse, zebrafish

## Abstract

Prostate cancers with mutations to a protein called SPOP use an error-prone method to repair broken DNA strands.

**Related research article** Boysen G, Barbieri CE, Prandi D, Blattner M, Chae SS, Dahija A, Nataraj S, Huang D, Marotz C, Xu L, Huang J, Lecca P, Chhangawala S, Liu D, Zhou P, Sboner A, de Bono JS, Demichelis F, Houvras Y, Rubin MA. 2015. *SPOP* mutation leads to genomic instability in prostate cancer. *eLife* **4**:e09207. doi: 10.7554/eLife.09207

**Image** Mutations to the SPOP protein (red) can lead to prostate cancer

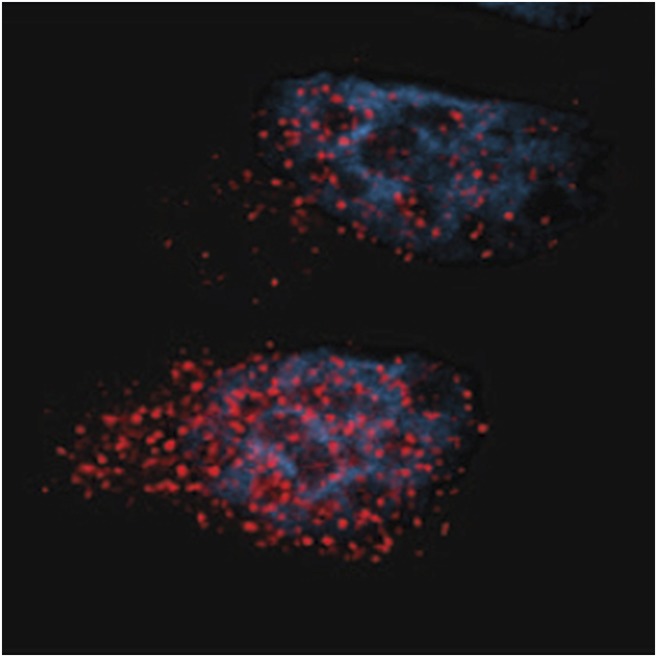

Changes to the genetic material of a cell can cause it to become cancerous. Recent data have demonstrated that extensive rearrangements of genetic material occur in prostate cancer (Berger et al., 2011; Baca et al., 2013). Generally, prostate tumors can be classified into those in which the rearrangement frequency is high or low. Now, in eLife, Mark Rubin of Weill Cornell Medical College and colleagues – including Gunther Boysen and Christopher Barbieri as joint first authors – shed light on why tumors with a mutation in a gene called *SPOP* have a high rearrangement frequency (Boysen et al., 2015).

Tumors with high rearrangement frequencies often have two genes deleted from their cells: the *MAP3K7* gene, which is deleted in 30–40% of tumors; and the *CHD1* gene, which is deleted in 15–20% of cancers (Liu et al., 2012). In prostate cancer, it is relatively rare to find mutations that affect single genes. However, recent large-scale genomic sequencing efforts have uncovered a few genes that are more often mutated than deleted or duplicated.

The most commonly mutated gene in prostate cancer encodes Speckle-type POZ protein (SPOP), which is mutated in around 10% of primary prostate tumors (Barbieri et al., 2012). In these tumors, mutations to the *SPOP* gene commonly occur alongside a loss of the *CHD1* and *MAP3K7* genes, and they are also associated with high numbers of genomic rearrangements. This has generally been attributed to the loss of the CHD1 protein. CHD4, a protein closely related to CHD1, directly interacts with DNA repair machinery (Pan et al., 2012), so it is widely assumed that CHD1 may also regulate DNA repair. However, there are currently no data to support this hypothesis.

Boysen, Barbieri et al. – who are based at Weill Cornell Medical College, the University of Trento and the Institute of Cancer Research in London – examined high-resolution genomic data from clinical prostate samples and found that SPOP mutations are strongly associated with high levels of genomic rearrangement. The *CHD1* and *MAP3K7* gene deletions were also equally and independently associated with large numbers of genomic rearrangements. However, an assessment of tumor clonality – the similarity of the genetic information found in different cells in the same tumor – suggested that the *SPOP* mutation occurred before the loss of either *MAP3K7* or *CHD1*. This supports the hypothesis that the SPOP protein helps to initiate the development of prostate tumors.

To uncover the molecular basis of this initiation, Boysen, Barbieri et al. used a zebrafish model to define how wild-type SPOP and a common SPOP mutant (called F133V) affect gene transcription. The data revealed that the presence of mutant SPOP causes an enrichment of genes that had previously been associated with mutant *BRCA1* – a gene that is mutated in some breast and ovarian cancers. The identity of the affected genes suggested that SPOP affects DNA repair pathways. Further investigation in human and mouse models confirmed that mutant SPOP blocks a process called called homology–directed repair: this is the method that cells normally use to repair double-stranded DNA breaks. The cells then have to rely on a less reliable repair method (the non-homologous end-joining pathway), and this increases the number of genomic rearrangements (Figure 1).

**Figure 1.**
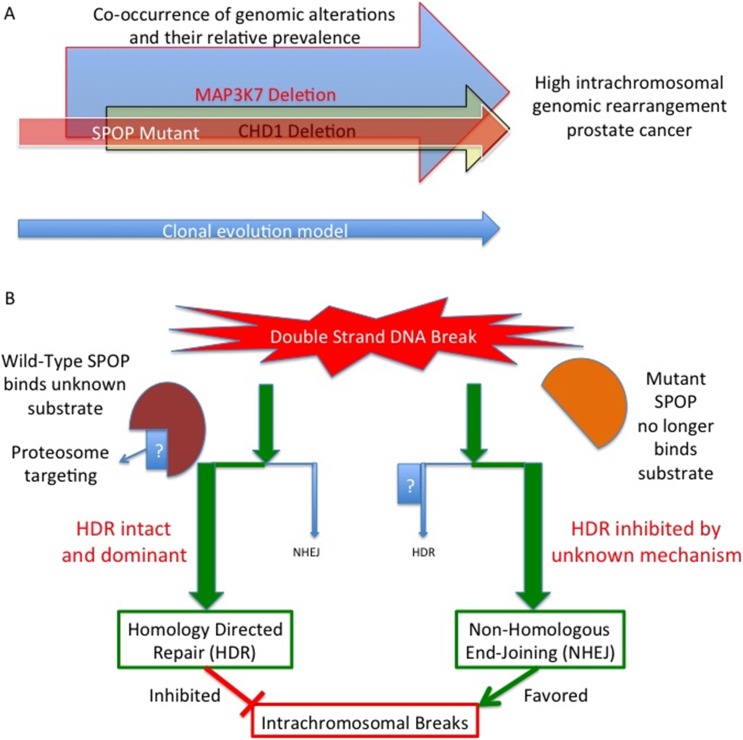
Mutant SPOP promotes genomic rearrangements within chromosomes. (**A**) SPOP mutation is an early event in a subtype of prostate cancer associated with a high genomic rearrangement frequency. The MAP3K7 and CHD1 proteins are also lost when SPOP mutations occur, and are each independently associated with high rearrangement frequencies. Based on a clonality model SPOP mutation preceeds MAP3K7 loss, which preceeds CHD1 loss. The frequency of loss for MAP3K7 (≅30%) is higher than that for CHD1 (≅15%) which is higher than the frequency with which SPOP mutation occurs (≅10%). (**B**) SPOP is an enzyme that enables homology-directed DNA repair (HDR) of double strand breaks. The substrate protein that is specifically involved in modulating repair is unknown. Mutant SPOP fails to promote HDR, and so the less stringent and more error prone non-homologous end joining (NHEJ) pathway becomes the favored repair pathway. This results in a high degree of intrachromosomal breaks and hence more genomic rearrangements.

Previous work has demonstrated that drugs that inhibit PARP (poly (ADP-ribose) polymerase 1), such as olaparib, can kill *BRCA1* mutant cancer cells, as well as other cells in which homology-directed repair does not work properly (Polyak and Garber, 2011). Boysen, Barbieri et al. therefore assessed whether *SPOP* mutant cells were also sensitive to olaparib, and found evidence that this is the case. This subtype of prostate cancer therefore has a unique sensitivity to PARP inhibition that could be immediately translated to clinical use.

Boysen, Barbieri et al. have provided key insight into how large numbers of genomic rearrangements occur in the aggressive SPOP/CHD1/MAP3K7 subtype of prostate cancer. However, additional studies are needed to establish further details about the specific pathways involved and to work out how the *SPOP* mutations interact with the loss of the *CHD1* and *MAP3K7* genes.

The SPOP protein targets various substrate proteins for degradation by adding a ubiquitin tag onto them. Known substrates of SPOP include the androgen receptor (An et al., 2014), the steroid co-activator SRC-3 (Geng et al., 2013), and the DEK and ERG oncogenes (Theurillat et al., 2014; An et al., 2015; Gan et al., 2015). All of these targets may affect the aggressiveness of prostate cancer. The specific target of SPOP in the context of DNA repair is not known and was not investigated by Boysen, Barbieri et al. However, all of these SPOP targets potentially interact with DNA repair processes, and there are many other identified SPOP targets with unknown roles that may produce the observed effects on the repair pathway. Future work will need to investigate this to provide more concrete mechanistic insight into the role of SPOP in modulating double-stranded DNA repair.

Loss of the *CHD1* and *MAP3K7* genes can also promote the development of prostate tumors in the absence of *SPOP* mutations (Wu et al., 2012; Rodrigues et al., 2015). In addition, they are both associated with enhanced genomic rearrangements when *SPOP* is intact, they are both highly clonal, and they both occur much more frequently than *SPOP* mutations. Modeling *SPOP* mutations in combination with *CHD1* and *MAP3K7* loss has not been reported; indeed, the specific roles of *MAP3K7* and/or *CHD1* loss in generating genomic rearrangements have not been explored. Given that CHD1 may affect DNA repair, and that the loss of the closely related CHD4 protein makes it easier for PARP inhibitors to kill cancer cells (Pan et al., 2012), such a model may provide mechanistic insights that focus future therapeutic approaches.
